# Characterization of annulus fibrosus lesions on magnetic resonance imaging in dogs affected by intervertebral disc disease, a descriptive case series

**DOI:** 10.3389/fvets.2024.1434447

**Published:** 2024-11-26

**Authors:** Yael Merbl, Sukhmeen Kaur, Tiffany G. Kei, Elle Ryan, Philippa J. Johnson

**Affiliations:** Department of Clinical Sciences, College of Veterinary Medicine, Cornell University, Ithaca, NY, United States

**Keywords:** high-intensity zone, MRI, canine, disc disease, annular fissure

## Abstract

**Objective:**

Describe and characterize the magnetic resonance imaging (MRI) appearance of annulus fibrosus (AF) high-intensity zone (HIZ) in dogs suffering from intervertebral disc disease (IVDD).

**Methods:**

A single-center retrospective case series study. Databases were reviewed from 2011 to 2022 for dogs that underwent MRI diagnosis due to suspected IVDD. Cases were included if they had T2-weighted (T2W) hyperintense annular fibrosus lesions (AFL) on the imaging diagnosis report. To be included, the MRI scan had to be of diagnostic quality and include a sagittal T2W, proton density (PD), or short tau inversion recovery (STIR) sequence of the annular lesion, together with transverse T2W and/or dorsal plane STIR sequences over the HIZ region.

**Results:**

Forty one cases (in 39 dogs) of HIZ were included in the study. Mixed breed dogs were the highest represented group representing 25.6% of the cohort. Patient median age was 7.5 years and median weight 23 kg. Primary HIZ appeared in 7/39 dogs (17.95%) and the remaining had acute non-compressive nucleus pulposus extrusion (ANNPE), hydrated nucleus pulposus extrusion (HNPE) or concurrent myelopathy. Characterization of HIZ lesions included several variable appearances in orientation and shape. HIZ lesions were most easily identifiable in the sagittal plane. Similar to humans, the most common site of HIZ without extrusion was the lumbosacral (LS) region. All the dogs with HIZ lesions as the most significant MRI finding, exhibited spinal pain and/or chronic paresis/plegia.

**Conclusions and clinical significance:**

By introducing and defining HIZ lesions to the veterinary imaging nomenclature, we hope future studies will further examine the prevalence and clinical significance of HIZ lesions in canine patients.

## 1 Introduction

Intervertebral disc disease (IVDD) is the most common cause for myelopathy in dogs (1) accounting for 3% of all cases presenting to emergency services in North America (2).

IVDD has been subclassified in dogs by the Canine Spinal Cord Injury Consortium (3). This classification subdivides intervertebral disc disease into Hansen type I/Acute IVD extrusion, Hansen type II/Chronic IVD protrusion, acute non-compressive nucleus pulposus extrusion (ANNPE) (4), hydrated nucleus pulposus extrusion (HNPE) (5, 6) and intradural/intramedullary intervertebral disc extrusion (IIVDE) (7) based on their MRI appearance. Each type of IVDD has unique clinical characteristics and carries a different prognosis, highlighting the importance of accurate and thorough characterization of this disease process. In humans, classifications of IVDD are well described and include lesions within the annulus fibrosus (AF) (8).

The AF comprises of concentric fibrocartilage lamellae. These lamellae are composed of elongated fibrocytes intermingled between collagen bundles with a network of elastin fibers found throughout (9, 10). In humans, lesions within the AF occur when there is fissuring or tearing of the annular fibers and have been subclassified into three forms; the concentric form describes a disruption between adjacent circumferentially layered short transverse fibers that form the annulus, the radial form describes a disruption of the longitudinal fibers through all layers of the annulus, and a third form known as a “rim lesion” describes the transverse rupture of fibers near their attachments with the ring apophysis. It has been suggested that the type 3 “rim lesion” annular fissures are more likely to cause pain than other forms (11, 12). On MRI, annular fissures appear as a high-intensity signal located in the dorsal (referred to as posterior) or ventral (anterior) annulus fibrosus surrounded by the low-intensity signal of the AF on T2-weighted (T2W) MRI, and as such, these lesions are referred to as “high-intensity zone” (HIZ) lesions (3, 9, 13–16). Controversy exists regarding the clinical importance versus incidental degenerative nature of HIZ in MRI studies. Several studies have debated the clinical importance of HIZ in relation to lower back pain (LBP) in humans (17, 18), while other studies reported an association between HIZ in the lumbar IVD and lower back pain and increased potential of LBP in humans (19), Another study researched the clinical significance of HIZ in relation to back pain and histopathological findings of annular tears in humans with HIZ present in their MRI (20). The researchers performed discography and tried to mimic the pain response of patients when injecting the contrast agent into the site of lesion. All 17 patients with discs with HIZ MRI finding had a painful response. On histology, abnormal morphology with annular tears reaching the outer third of the AF were noted and when assessing sagittal slices through the HIZ lesions, a formation of vascularized granulation tissue in the outer region of the annulus fibrosus was reported.

High signal annular fibrosus lesions (AFL) have been previously recognized in canine IVDD in both ANNPE and HNPE forms of IVDD (9, 10) as small tears that occur following acute changes in intradiscal pressure (21, 22). AFL are also described histologically in canine IVDD in areas where chondrocyte metaplasia and tears with cleft formation are documented (23). AFL lesions independent of intervertebral disc extrusion are a relatively common finding in canine spinal MRI in the authors clinical experience. These lesions, at times, have been associated with spinal pain and have not been described in canine. Additionally, focused evaluation and classification of annular lesions on MRI have not been performed in the dog despite their instrumental involvement in certain forms of IVDD and suspected clinical significance. In this retrospective case series study, we performed a focused evaluation of AFL showing HIZ in a cohort of canines. The goal of this study is to document the signalment and clinical characteristics of these HIZ lesions and to describe their morphology and appearance on MRI. By characterizing and assessing AFL lesions on MRI in the canine, we provide a foundational step to improve our understanding of these lesions and their clinical significance.

## 2 Materials and methods

### 2.1 Case selection

The aim of case selection was to identify a cohort of dogs with intervertebral discs with HIZ lesions. In order to do this we searched for dogs that had undergone MRI of the vertebral column and had an imaging diagnosis or description indicating the presence of annular trauma, tearing, or fissure formation (9, 10). Because T2-weighted hyperintense annular lesions had been previously described in ANNPE and HNPE forms of IVDD our search crieteria included cases with an imaging diagnosis of ANNPE, HNPE as well as any case that had an imaging diagnosis or description that included annular rupture, tear, fissure or HIZ. To identify these cases, a previously collated spreadsheet documenting the imaging diagnosis of all MRIs obtained at Cornell University College of Veterinary Medicine Small Animal Hospital between 2011 and 2018 was searched. In addition, to identify cases obtained from 2019 to 2022 the imaging reporting system was searched. Both data resources were searched for key words in the imaging diagnosis. Key words searched included “Acute non-compressive nucleus pulposus extrusion,” “hydrated nucleus pulposus extrusion,” “traumatic disc,” “discal cyst,” “annular fissure,” “annular tear,” “annular trauma,” “annular rupture,” “high-intensity zone,” “HIZ” and “hyperintensity zone.” This initial search identified 112 cases.

Each of these cases were reviewed by a trained rotating intern (SK) and a board-certified radiologist (PJJ) and included if they met the following inclusion criteria:

1. Had a T2W high-intensity annular lesion (HIZ lesion) within the dorsal annulus of atleast one IVD.
2. The MRI scan was high diagnostic quality without significant artifact.
3. The MRI study included a sagittal T2W, proton density (PD), or short tau inversion recovery (STIR) and a transverse T2W and/or dorsal plane STIR sequence over the affected IVD (s).

This evaluation identified 39 cases with at least one HIZ lesion. Two cases exhibited two discs with HIZ lesions.

### 2.2 Clinical and imaging information

For each case signalment including breed, age, sex, and reproductive status (intact vs. altered) were collated. The clinical records were reviewed for each to determine their neurological presentation, which was recorded. Imaging information including MRI date, sequences included and imaging diagnosis were recorded.

### 2.3 HIZ characterization

**Figure 1:**
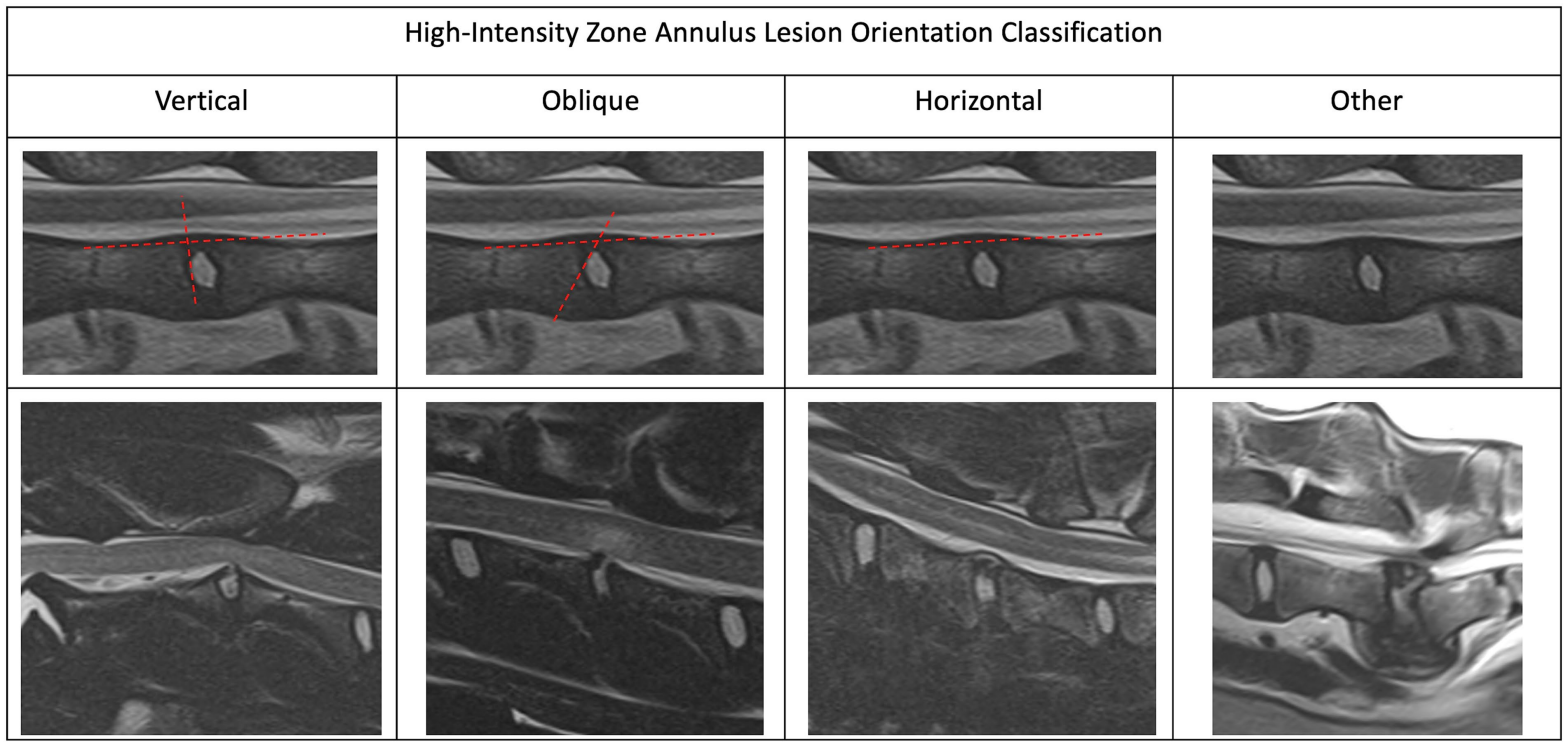
Magnetic resonance imaging. Sagittal plane images depicting the different high-intensity zone orientations. We characterized the HIZ orientation as vertical, oblique, horizontal or other.

**Figure 2:**
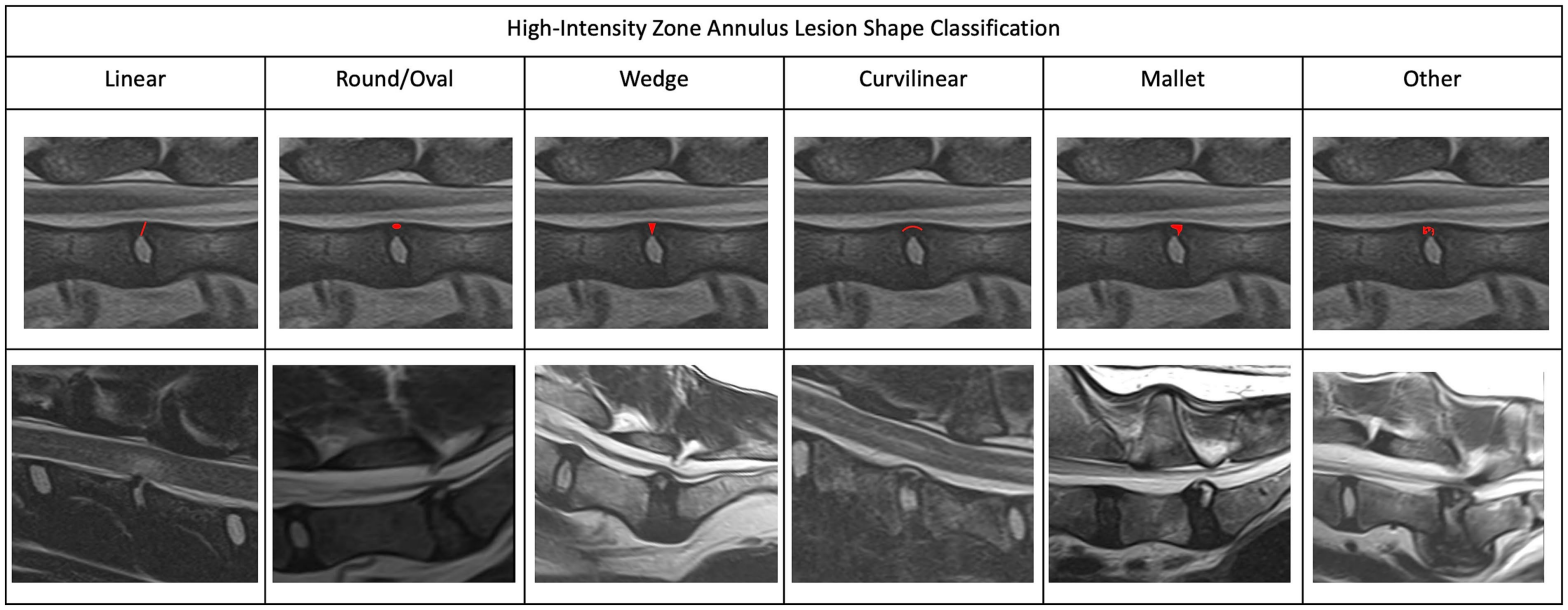
Magnetic resonance imaging. Sagittal plane images depicting the different highintensity zone shape classification. We characterized the HIZ shapes as linear, round/oval, wedge, curvilinear, mallet, or other.

**Figure 3:**
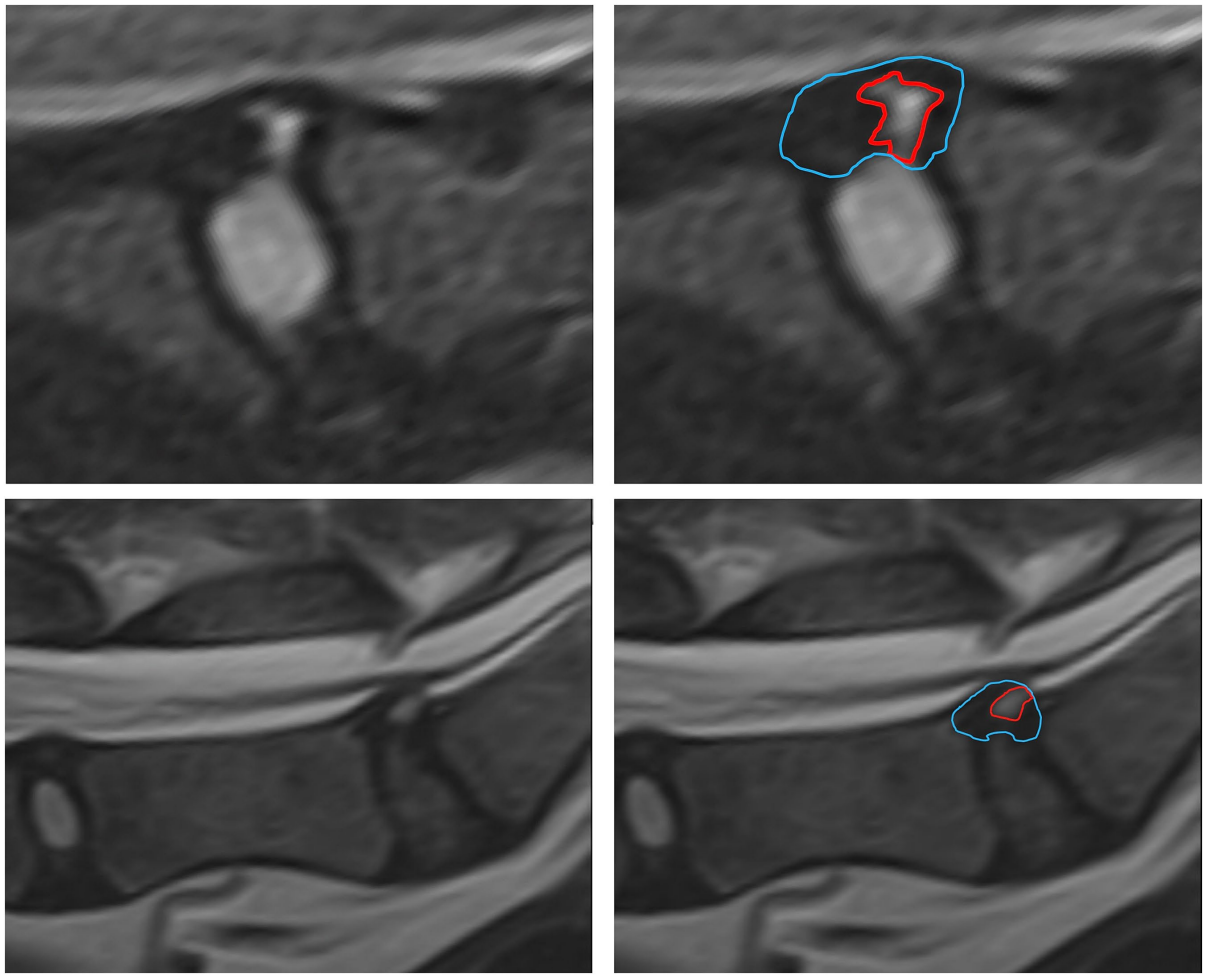
Measurements of the HIZ cross sectional area and annulus area were performed using Picture Archiving and Communication Systems (PACS) on diagnostic display devices are shown.

**Figure 4:**
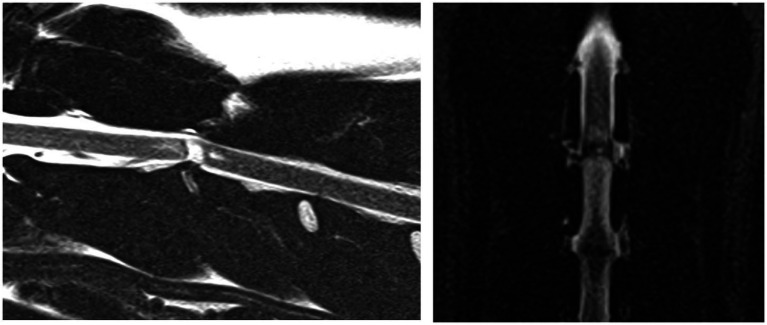
A case example demonstrating a case where the annular lesion was visible on both the sagittal and dorsal plane images. Sagittal T2-weighted and dorsal STIR images of the cervical vertebral column (C2-C4). This annular lesion was at C2-3 and was classified as linear, oblique and present within the caudal aspect of the dorsal annulus. This lesion was associated with a final diagnosis of ANNPE.

In order to create a useful characterization of HIZ lesions a small cohort of HIZ cases were evaluated by the investigators. Shape and form terminology described in the human literature was used as a guide (14, 24) and additional terms were added to allow for the most appropriate classification in our canine cohort. The final HIZ classification included the following descriptors: vertebral location of the disc, lesion orientation classification (vertical, oblique, horizontal, or other) (Figure 1) and lesion shape classification (linear, round/ oval, wedge, curvilinear, mallet, or other) (Figure 2). Additionally, location of the HIZ within the annulus in sagittal plane (cranial, middle, caudal, diffuse, or multifocal), location of HIZ in transverse plane (sagittal, right, or left parasagittal), lesion area (mm^2^), annulus area (mm^2^) and visibility on transverse or dorsal plane images (when available) were recorded. Measurements were not performed on cases where HIZ lesion margins were not well defined, preventing accurate measurement. Measurements of the HIZ lesion and annulus areas were performed using Picture Archiving and Communication Systems, (Philips, 12.2, USA, 2022), on diagnostic display devices as shown in Figure 3. Concurrent disc information was documented and included the presence or absence of disc herniation, reduction in the volume and intensity of the nucleus pulposus (NP) (compared to adjacent discs), cord compression, cord lesions, and epidural disc material. Concurrent abnormalities present within the vertebral column were also recorded (Figure 4).

**Figure 5:**
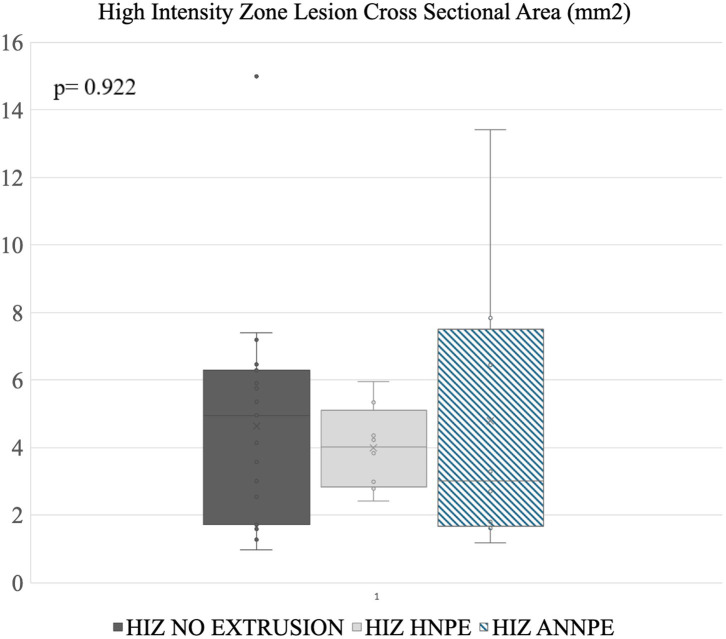
Disc lesion cross sectional area measurements. The line within a box indicates the median, X indicates the average. The lower and upper lines indicate 25th and 75th percentiles of data (i.e., interquartile range, IQR) respectively. No significant differences in surface area were noted between the different groups..

### 2.4 Statistical analysis

Annular cross sectional lesion size was analyzed and visualized using a commercially available statistical analysis software Excel 2021, (Microsoft Corporation, 2020, USA) and JMP Pro 17, SAS Institute Inc., Cary, NC, 1989–2023. A Kruskal-Wallis test was used to test whether the groups had different median HIZ cross sectional lesion area, because groups had different variances. The results are presented as the median (interquartile range IQR). A *p*-value of <0.05 was considered statistically significant (Figure 5).

## 3 Results

Thirty nine dogs exhibited a HIZ within the dorsal annulus in at least one of their intervertebral discs. Two dogs exhibited two intervertebral discs with HIZ lesions, meaning that our cohort included 41 affected intervertebral discs. These HIZ were associated with an ANNPE in 9 discs, an HNPE in 9 discs, and represented a HIZ without extruded material in 23 discs.

The signalment and clinical evaluation of all cases is listed in Table 1.

**Table 1:** Signalment and clinical evaluation of high-intensity zone cases divided into the study groups.

Group		# of Hiz lesions noted	Age months Mean (SD)	Females	Males	Body weight Kg Mean (SD)	Clinical signs					Clinical course	
							Plegia	Non-ambulatory paresis	Ambulatory paresis	Pain	Other	Acute	Chronic
HIZ without NP extrusion	Primary HIZ	7	106.3 (30.6)	3	4	22.6 (9.0)	1	3	2	1	0	4	3
	HIZ with concurrent pathology	16	85.3 (27.5)	6	10	26.8 (10.0)	6	6	3	0	1	15	1
HIZ associated with ANNPE		9	76.4 (26.0)	5	4	20.1 (9.8)	2	6	1	0	0	9	0
HIZ associated with HNPE		9	102.3 (26.7)	7	2	23.2 (9.0)	2	7	0	0	0	8	1

HIZ, high-intensity zone; NP, nucleus pulposus; ANNPE, acute non-compressive nucleus pulposus extrusion; HNPE, hydrated nucleus pulposus extrusion; SD, standard deviation.

### 3.1 Characterization of HIZ associated with ANNPE

Nine dogs exhibited a HIZ within the dorsal annulus with concurrent suspected ANNPE. This included one dog from each of the following breeds: Labrador Retriever, English Bulldog, Pomeranian, Yorkshire Terrier, Staffordshire Bull Terrier, Shetland Sheepdog, Boxer, and 2 mixed breed dogs. Age of affected individuals ranged between 20 and 119 months. Dogs presented with either hemi-, para-, or tetra-paresis/plegia.

Six of 9 dogs had the HIZ lesion in the cervical region: C4-5 (*n* = 2), C2-3 (*n* = 1), C3-4 (*n* = 1), C5-6 (*n* = 1), and C6-7 (*n* = 1). Within the cervical vertebral column lesions, the orientations of the lesions were oblique (*n* = 3), vertical (*n* = 2) and other (*n* = 1). HIZ shapes included wedge (*n* = 1), linear (*n* = 1), round (*n* = 1), mallet (*n* = 1) or other (*n* = 2). Most lesions affected the caudal annulus (*n* = 4), and the remaining were cranially and multifocally distributed (*n* = 1 each). 3/9 dogs had the HIZ lesion in the thoracolumbar spine: T9-10 (*n* = 1), T12-13 (*n* = 1), and L2-3 (*n* = 1). Within the thoracolumbar vertebral column, all lesions were other in orientation and lesion location in the dorsal annulus were multifocally (*n* = 2) and cranially (*n* = 1). HIZ shapes included wedge (*n* = 1), and Other (*n* = 2).

HIZ was present at the mid-sagittal level, for majority of cases (*n* = 7), with only two cases being parasagittal. The visibility of the HIZ lesions was noted in 5 of the 9 cases in the dorsal plane STIR, and in all of the T2W transverse images.

HIZ cross sectional lesion area was most variable in this group, with a median of 3 mm^2^ [Reference range (RR): 1.17–13.41 mm^2^] (Figure 5).

### 3.2 Characterization of HIZ associated with HNPE

Nine dogs exhibited HIZ lesions within the dorsal annulus with concurrent suspected HNPE. This included Doberman Pinchers (*n* = 2) and one dog of each of the following breeds: Cardigan Welsh Corgi, German Short-haired Pointer, Basset Hound, Lhasa Apso, Yorkshire Terrier, English Springer Spaniel and a mixed breed dog. In this cohort, dogs presented with ages ranging between 48 and 144 months. Dogs presented with mainly hemi- or tetra-paresis/plegia; only one dog was paraparetic on presentation, and for two dogs clinical presentation was unavailable.

Six of 9 HIZ lesions were in the cervical spine: C5-6 (*n* = 4), and C4-5 (*n* = 2); Within the cervical spine, the orientation of the lesions tended to be oblique (*n* = 5) and 1 dog had a vertical orientation. In all 6 dogs the caudal annulus was affected (*n* = 6). 3/9 HIZ lesions were in the thoracolumbar spine: T13-L1 (*n* = 2), and T12-13 (*n* = 1). These lesions were more variable in orientation and shape than observed in the cervical spine. Lesions were oblique (*n* = 1), horizontal (*n* = 1), and other (*n* = 1) in orientation, curvilinear (*n* = 1) and other (*n* = 2) in shape, and affected either the caudal annulus (*n* = 1) or the annulus diffusely (*n* = 2).

The HIZ was most visible on either the sagittal (*n* = 8) or right parasagittal (*n* = 1) plane image. In addition to the T2W sagittal appearance, the visibility of the HIZ lesions was noted in 1 of the 9 cases in T1- weighted (T1W), dorsal STIR recovery and in 4 of T2W transverse images. The remaining HIZ cases were not noted in these planes and only in the sagittal plane, with a median cross sectional area measuring 4.03 mm^2^ (RR: 2.42–5.95 mm^2^) (Figure 5).

### 3.3 Characterization of HIZ without NP extrusion

Twenty-three dogs exhibited lesions within the dorsal annulus that was not associated with extruded material from that intervertebral disc. This subgroup included mixed breed dogs (*n* = 8), Labrador retrievers (*n* = 3), Shih Tzus (*n* = 2), and one of each of the following breeds: Chihuahua, Australian Shepherd, Bulldog, Springer Spaniel, Yorkshire Terrier, Golden Retriever, Standard Poodle, German Short-haired Pointer and German Shepherd, and Doberman Pincher. Age of affected individuals ranged between 28 and 152 months. Dogs presented with a various clinical signs ranging from neck hyperesthesia to loss of deep pain nociception.

In sixteen of the HIZ cases, a significant concurrent pathology was noted in a different location and suspected as the cause of clinical signs. Of those, 7 cases had an ANNPE, 3 had a HNPE, 1 had an IIVDE, 2 had spinal tumors, 2 had a suspected myelitis, 1 had suspected fibrocartilogenous emboli. In the 7 remaining cases, HIZ was the only significant lesion identified. Of those without concurrent disease, 2 dogs had a HIZ in the cervical vertebral column (C3-4, C6-7) and exhibited neck pain, and five had their HIZ in the lumbosacral region at L7-S1 (*n* = 3), L6-7 (*n* = 1) and L3-4 (*n* = 1) and exhibited chronic ambulatory or non-ambulatory paraparesis /plegia.

More HIZ lesions without extrusion were located in the thoracolumbar or lumbosacral vertebral column (*n* = 16) than the cervical vertebral column (*n* = 7). In the thoracolumbar vertebral column, most HIZ lesions without extrusion were located at the L7-S1 intervertebral disc space (*n* = 10) and the rest at L6-7 (*n* = 2), L3-4 (*n* = 2), L2-3 (*n* = 1), and T13-L1 (*n* = 1). In the cervical vertebral column, no preferred HIZ location was noted and lesions were present at C2-3 (*n* = 2), C5-6 (*n* = 2), C6-7 (*n* = 2) and C3-4 (*n* = 1).

HIZ lesion orientation was variable with being categorized as horizontal (*n* = 8), oblique (*n* = 9) other (*n* = 4), and 2 lesions were identified as vertical.

Within the cervical vertebral column, the orientation of the lesions tended to be horizontal and diffuse (*n* = 3) or oblique and caudal (*n* = 3) and 1 dog exhibited horizontal and caudal. Variable lesion shapes were noted including linear (*n* = 2), curvilinear (*n* = 2), oval (*n* = 1), mallet (*n* = 1), and other (*n* = 1).

In the thoraco-lumbo-sacral vertebral column, the lesions were oblique (*n* = 6), horizontal (*n* = 4) vertical (*n* = 2) or other (*n* = 4) in orientation and affecting the caudal annulus (*n* = 9), cranial annulus (*n* = 5), diffuse (*n* = 1) and multifocal (*n* = 1). Lesions were variable in shape including round/oval (*n* = 5), wedge (*n* = 3), other (*n* = 4), linear (*n* = 2), curvilinear (*n* = 1), and mallet (*n* = 1).

HIZ lesion location within the annulus in sagittal plane was caudal (*n* = 13), diffuse (*n* = 4), multifocal (*n* = 1) and cranial (*n* = 5). The sagittal plane was most useful in recognizing the HIZ (*n* = 19) and in the 4 remaining cases, the HIZ was apparent in the right parasagittal plane. The visibility of the HIZ lesions was noted in 5 of the 23 cases in T1W dorsal STIR recovery, and in 6 of the 23 cases in T2W transverse images. The remaining HIZ cases were not noted in these planes and only in the sagittal plane.

HIZ lesions found in the MRI studies without NP extrusion had a median cross sectional area of 4.96 mm2 (RR: 1.59–14.99 mm^2^) (Figure 5).

### 3.4 HIZ Cross Sectional Area

39 HIZ lesions were measured. In two cases HIZ lesion margins were not well defined, preventing measurements. HIZ lesions in the ANNPE group had a median cross sectional area of 3 mm^2^ (RR: 1.17–13.41 mm^2^). The HNPE group had a median cross sectional surface area of 4.03 mm^2^ (RR: 2.42–5.95 mm^2^) and the HIZ without extrusion group had a cross sectional surface area of 4.96 mm^2^ (RR: 1.59–14.99 mm^2^)No significant differences in lesion area were identified between groups (*p* = 0.922) suggesting that lesions were not significantly different when associated with intervertebral disc displacements (Figure 5).

## 4 Discussion

This study aimed to provide a complete clinical and morphological evaluation of HIZ lesions within the AF of dogs with IVDD. We collated MRI cases that were diagnosed with an AF lesion over an 11 year period at the Cornell University Hospital for Animals. These cases included HIZ lesions with associated HNPE or ANNPE and primary HIZ lesions with and without significant pathology at a distant site. We documented the signalment and clinical status of the subjects. On MRI, we classified the morphological characteristics of the HIZ lesions according to location within the vertebral column as well as shape, orientation, and position within the dorsal annulus. We determined that HIZ lesions associated with some extrusion types often had a common morphology; however, lesion size was not significantly different between groups. Clinically, when HIZ lesions were the only significant MRI findings, pain or chronic paresis were present. This finding warrants future studies as the lesions may hold clinical importance.

In our cohort we found HIZ annular lesions associated with disc extrusions (HNPE and ANNPE) and those not associated with disc extrusions (primary HIZ lesions). Within the primary type HIZ lesion group, 16 dogs had clinically significant lesions elsewhere within the nervous system, but in 7 cases the HIZ lesion was the most significant pathology identified on this examination. By documenting the morphology of the HIZ lesions and correlating this to the underlying diagnosis, we found that some forms of the condition trended toward having a standard morphology. For instance, in HNPE lesions of the cervical vertebral column, the HIZs tended to affect the caudodorsal annulus and be linear or wedge shaped with an oblique orientation. This pattern is likely to reflect the relationship between forces sustained in the cervical vertebral column and the anatomic factors predisposing some regions of the annulus to tearing.

The terminology used in human medicine differs from the terminology we believed would best describe our findings. In humans, HIZs are described as: round, fissure, vertical, rim, and enlarged in one study, and additional descriptions such as “rat tail” and “mallet head” are described in another (14, 24). Our terminology aimed to create easy identification for veterinary radiologists and addressed the need for more specific differentiation based on the different lesion types noted in this cohort. A round shape was considered the most common lesion shape in humans, whereas in dogs, only the lumbar and sacral regions were more likely to show a round shape, and the cervical and thoracic spine showed a much more variance in lesion shape. Fissure shapes identified in humans HIZ lesions correlated well with linear and wedge shapes of this study. Additionally, in only 4.6% of cases, the HIZ were identified in axial view (18), which is similar to our finding that HIZs were also less detectable on the transverse plane.

Similar to HIZ in humans, the most common site of HIZ without extrusion was the lumbosacral region. The reason for preferential distribution of HIZs to the lumbosacral region is yet to be determined, however it may be related to the unique anatomy and biomechanical forces sustained in this region. In the dog, the L7-S1 intervertebral disc is the largest disc in the spine and the lumbosacral junction is the most dynamic region of the thoracolumbosacral vertebral column able to rotate, bend, flex and extend. These factors are thought to predispose this region to intervertebral disc degeneration, which may contribute toward annular tearing or fissure formation (25, 26).

In humans, some AFL can be asymptomatic, while others have been associated with chronic pain. Moreover, AFL have been suggested to contribute to disc extrusions at a later time (27). Similar to humans, it is possible that HIZ lesions in dogs may be clinically incidental, however this would require evaluating the MRIs of neurologically normal dogs to determine if HIZ lesions can be found in clinically normal dogs. Humans and dogs share many similarities in the pathophysiology of degenerated discs (28) however contrary to our findings, in humans with HIZ lesions, the most common presenting complaint is lower back pain, with severe neurological deficits not being a common feature (28). This difference may be related to the differences in spinal canal axis, anatomy, and biomechanical forces; for instance, in humans, disc material can extrude in either a posterior or anterior direction, and in many cases will affect the nerve roots (29) running in a region of the lower spine where the spinal cord has already terminated. In dogs, the disc preferentially extrudes in an posterior (dorsal) direction, and commonly the disc extrudes/protrudes and subsequently compresses the spinal cord directly, causing more severe neurological deficits (30).

In our evaluation, we utilized the sagittal plane T2-weighted image to screen for HIZ lesions. Screening for lesions in such a way is common practice in veterinary radiology, with the T2-weighted image often directing where orthogonal slices are placed. Despite this being common practice, this approach does bias our results as HIZs are small, focal lesions and may be overlooked on the sagittal plane if they are more lateralized. Additionally, as primary HIZ lesions have yet to have been described in the veterinary literature, it is possible that small HIZ lesions may have been considered clinically incidental by the reviewing radiologist. For this reason, some cases may not have transverse images performed over the HIZ lesions, limiting our study.

Of the 23 cases of primary HIZ lesions, 5 were mixed breed dogs and 8 dogs were among breeds recommended for testing of CDPA/CDDY phenotype variants, markers for chondrodysplasia and chondrodystrophy with links to premature intervertebral disc degeneration (22). We cannot draw any conclusions related to signalment of dogs presenting with HIZ due to the small number of cases meeting the inclusion criteria. Future studies should include a larger sample size and try to associate the age and size of dogs as well as chondrodystrophic versus non-chondrodystrophic status with the prevalence of HIZ lesions and the tendency toward developing certain AFL phenotypes.

The etiology and pathophysiology of HIZ are still not well understood (31). In humans, harvested HIZ discs were evaluated histologically and newly formed blood vessels were identified around the tears. The authors hypothesized the HIZ lesion might have been the result of disorganized, vascularized granulation tissue, formed as a part of the healing response to an annular tear (32). It was suggested that the high stress between the laminar layers originating from asymmetric disc morphology could play a role in further disc degeneration and laminar separation of annulus fibers that may contribute to the increased prevalence of HIZ lesions directed toward the posterior side (32). Future studies focusing on AFLs in dogs should explore the relationship between the size/shape and timing of apprearnace of HIZ lesions in MRI related to the insult, and spinal hyperesthesia in dogs, as well as delve into histopathological analysis of AFLs and their correlation to appearance on MRI.

With MRI technology increasing in availability and a more detailed classification system, future large-scale population studies may contribute to our knowledge on the degree of involvement in HIZs in disc degeneration and chronic pain in the veterinary population. We speculate that HIZ lesions are both underdiagnosed and underreported. This is likely due to a lack of understanding toward the significance of these lesions as well as their small size and limitations of the spatial resolution in MRI. Growing awareness of this MRI finding may help improve recognition and it’s documentation in the veterinary field. Additionally, employing more advanced pulse sequences in the evaluation of these lesions would improve their visibility and allow for more accurate clinical evaluations. High resolution proton density and T2-weighted sequences performed on high field MRI have provided excellent detail of annular lesions in intervertebral disc disease and optimized sequences such as these could be utilized in both the clinical and research settings (33). The morphology and topography of HIZs could also be investigated in a larger scale longitudinal study to further elaborate the role of HIZ in the pathophysiology of canine IVDD.

## 5 Study limitations

Due to the retrospective nature of this case series, study limitations included low case number, a heterogenous study population, absence of a control group, and absence of standardized imaging descriptions, the latter of which may have enabled us to increase study sample size. In this study, we aimed to characterize cases demonstrating HIZs on MRI that were recorded either as an incidental finding or suspected to be related to the cause of clinical signs. Future research should aim to investigate the prevalence of HIZs in dogs through longitudinal data collection including clinical descriptions such as body score condition and neutering status to establish the clinical importance of HIZ in dogs with predisposed phenotypes.

## Data Availability

The raw data supporting the conclusions of this article will be made available by the authors, without undue reservation.

## Supplementary material

SUPPLEMENTARY TABLE 1 Data sheet of the study excluding the medical record number and DOB for protection of pet privacy.
